# Protein Expression Profiles of Antiseptic-Adapted *Escherichia coli*

**DOI:** 10.3390/microorganisms14071533

**Published:** 2026-07-14

**Authors:** David L. Auer, Uemmuehan Akyol, Denise Muehler, Konstantin J. Scholz, Karl-Anton Hiller, Tim Maisch, Wolfgang Buchalla, Ali Al-Ahmad, Fabian Cieplik

**Affiliations:** 1 Department of Operative Dentistry and Periodontology, Center for Dental Medicine, Medical Center & Faculty of Medicine, University of Freiburg, 79106 Freiburg, Germany; david.auer@uniklinik-freiburg.de (D.L.A.); konstantin.scholz@uniklinik-freiburg.de (K.J.S.); karl-anton.hiller@klinik.uni-regensburg.de (K.-A.H.); ali.al-ahmad@uniklinik-freiburg.de (A.A.-A.); 2 Department of Conservative Dentistry and Periodontology, University Hospital Regensburg, 93053 Regensburg, Germanywolfgang.buchalla@ukr.de (W.B.); 3 Department of Dermatology, University Hospital Regensburg, 93053 Regensburg, Germany; tim.maisch@klinik.uni-regensburg.de

**Keywords:** antiseptics, CHX, resistance

## Abstract

Repeated exposure to subinhibitory concentrations of antiseptics may lead to reduced susceptibility or resistance and potentially promote cross-resistance to antibiotics. However, the underlying molecular mechanisms remain incompletely understood. This study investigated whether antiseptic-adapted Escherichia coli strains exhibit altered protein expression profiles compared with wild-type (WT) *E. coli*. Protein expression was analysed in *E. coli* strains previously adapted over ten passages to subinhibitory concentrations of chlorhexidine (CHX), cetylpyridinium chloride (CPC), and benzalkonium chloride (BAC). WT bacteria were exposed to sub-minimum inhibitory concentrations (sub-MICs) of antiseptics for 3 h to induce stress. Untreated WT and heat-shocked WT *E. coli* (42 °C, 2 h) served as controls. Protein expression profiles were assessed using SDS-PAGE and Western blotting targeting stress-associated proteins. SDS-PAGE demonstrated altered protein expression patterns in antiseptic-adapted strains, including differences in band intensities and additional protein bands. Western blot analysis showed increased DnaK and GroEL expression with reduced LexA levels in heat-shocked WT bacteria, whereas RecA remained largely unchanged. Antiseptic-adapted strains exhibited increased DnaK, GroEL, and OmpF expression together with reduced LexA and RecA expression. These findings indicate that adaptation to antiseptics involves complex mechanisms among multiple proteins rather than a single adaptive mechanism.

## 1. Introduction

The increasing prevalence of resistant microorganisms reduces the effectiveness of antimicrobial therapies and represents a major global public health challenge. According to the Global Burden of Disease (GBD) 2021 study, bacterial antimicrobial resistance (AMR) was associated with an estimated 4.71 million deaths worldwide, including 1.14 million deaths directly attributable to bacterial AMR in 2021 [1]. Among infectious diseases, lower respiratory tract infections accounted for the largest proportion of AMR-associated deaths, with approximately 1.64 million associated and 0.40 million attributable deaths, highlighting the substantial contribution of bacterial pathogens such as Escherichia coli to the global AMR burden [2]. While antibiotic misuse has traditionally been considered the primary driver of AMR, there is growing consensus that non-antibiotic antimicrobial agents, including antiseptics and disinfectants like chlorhexidine digluconate (CHX) and cetypyridinium chloride (CPC), also contribute to resistance development when used inappropriately, for instance following insufficient indication or inadequate concentrations or durations leading to sublethal exposure [3,4,5]. In this context, the International Association of Dental, Oral and Craniofacial Research (IADR) has emphasized the responsibility of oral health research and clinical practice in addressing AMR, highlighting the need to better understand the biological consequences of frequent antiseptic use and to promote antimicrobial stewardship in dentistry [6].

Antiseptics are extensively used in dentistry, healthcare settings, and over-the-counter consumer oral hygiene products due to their broad antimicrobial activity [4,5,7]. However, increasing evidence indicates that repeated exposure to sub-minimum inhibitory concentrations (sub-MICs) of these agents can induce bacterial stress responses and promote adaptive phenotypes rather than complete microbial eradication [8,9,10]. Experimental studies have demonstrated that sub-MIC exposure to antiseptics may result in phenotypic adaptation to these antiseptics, altered membrane permeability, activation of efflux mechanisms, and, in some cases, cross-resistance to clinically relevant antibiotics [4,8,11,12,13,14,15].

Commercial antiseptic formulations are generally applied at concentrations that exceed the minimum inhibitory concentrations (MICs) of susceptible bacteria by several orders of magnitude [16]. However, dilution by biological fluids, adsorption to organic material, and declining antiseptic concentrations during application may result in residual concentrations within or below the MIC range, thereby exposing bacteria to sub-inhibitory antiseptic concentrations capable of inducing adaptive responses [16,17]. In contrast to antibiotics, standardized concepts such as the mutant prevention concentration (MPC) and the mutant selection window (MSW) have not yet been established for antiseptics, and consensus definitions, epidemiological cut-off values, and clinical breakpoints are currently lacking for CHX, CPC, and benzalkonium chloride (BAC) [18].

Repeated exposure to antiseptics within the oral environment may potentially contribute to the oral cavity becoming a reservoir of AMR genes, facilitated by the complexity of oral biofilms and the risk of horizontal gene transfer [14,15,19]. Previous investigations have shown that oral bacteria subjected to prolonged subinhibitory antiseptic stress exhibit phenotypic adaptation characterized by increased MICs, enhanced stress tolerance and altered biofilm behaviour including changes in biofilm biomass, composition, and susceptibility to antimicrobial agents [12,20]. Such adaptations may not only compromise antiseptic efficacy but also contribute to the broader AMR reservoir, as stress-induced responses can increase mutation rates and facilitate genetic and phenotypic diversity [21,22]. At the molecular level, bacterial adaptation to environmental stress is mediated by complex regulatory networks involving stress response proteins, chaperones, DNA repair systems, and membrane-associated proteins [3,23,24]. Proteins such as DnaK and GroEL play central roles in maintaining protein homeostasis under stress, while regulatory proteins including LexA and RecA coordinate DNA damage repair and SOS response, a conserved regulatory network activated upon DNA damage [25,26]. Alterations in outer membrane proteins, such as OmpF, may further decrease outer membrane permeability, leading to reduced intracellular antimicrobial accumulation, decreased antimicrobial susceptibility and consequently increased bacterial survival [27]. Despite these insights, systematic analyses of changes associated with long-term antiseptic adaptation on the protein level have not yet been comprehensively investigated. The *E. coli* strains investigated in the present study originated from our previously established adaptation experiments, in which repeated exposure to sub-minimum inhibitory concentrations (sub-MICs) of CHX, CPC and BAC resulted in stable phenotypic adaptation [20]. Among the investigated bacterial species, *E. coli* was the only organism for which stable adaptation could be generated for all three antiseptics, providing a well-characterized model to compare antiseptic-specific stress responses. Furthermore, *E. coli* was selected due to its well-characterized stress response systems and the availability of specific antibodies for protein-level analyses. In addition, previous work demonstrated phenotypic adaptation to antiseptics in *E. coli*, making it a suitable model to investigate underlying molecular mechanisms [20].

Therefore, the present study aimed to investigate protein expression profiles in *E. coli* strains that were previously subjected to ten passages of culture in subinhibitory concentrations of CHX, CPC, or BAC [20]. By comparing protein expression profiles of antiseptic-adapted strains with wild-type controls using SDS-PAGE and targeted Western blot analyses, this work seeks to investigate signatures of antiseptic adaptation on the protein level and to contribute to a more comprehensive understanding of how repeated antiseptic exposure may influence bacterial stress responses.

## 2. Materials and Methods

### 2.1. Bacterial Strains and Culture Conditions

Wild-type (WT) *E. coli* (ATCC 25922) served as the parental strain. WT cultures were streaked onto Müller–Hinton agar and incubated for 18–24 h at 37 °C. Single colonies were inoculated into 5 mL Müller–Hinton broth (MH; Merck KGaA, Darmstadt, Germany) and grown overnight at 37 °C under shaking conditions.

### 2.2. Determination of MICs and Sub-Minimum Inhibitory Concentrations (Sub-MICs)

*Escherichia coli* ATCC 25922 wild-type (WT) and the previously established antiseptic-adapted strains CHX-P10, CPC-P10, and BAC-P10 were used in this study. The adapted strains originated from our previous work, in which stable phenotypic adaptation was induced by repeated exposure to sub-MICs of CHX, CPC and BAC over ten consecutive daily passages [20]. During each passage, bacteria were incubated for 24 h in the presence of the respective antiseptic. The minimum inhibitory concentration (MIC) was defined as the lowest antiseptic concentration that completely inhibited visible bacterial growth after overnight incubation, whereas the sub-minimum inhibitory concentration (sub-MIC) was defined as the highest antiseptic concentration that still permitted visible bacterial growth, assessed by turbidity. Following each passage, bacteria from the sub-MIC well were transferred into fresh antiseptic-free Mueller–Hinton (MH) broth, incubated overnight, and subjected to a new MIC determination. This procedure was repeated for ten consecutive passages. Thereafter, the adapted strains were cryopreserved at −80 °C, and the stability of the phenotypic adaptation was confirmed by repeated MIC determinations following growth in antiseptic-free medium.

For the present study, WT and adapted strains were recovered from frozen samples and cultured overnight in MH broth at 37 °C under aerobic conditions. Before the stress exposure experiments, MIC and sub-MIC values for CHX, CPC, and BAC were re-determined using a broth microdilution assay. Twofold serial dilutions of each antiseptic, starting at 256 µM, were prepared in 48-well microtiter plates (Greiner Bio-One International GmbH, Kremsmünster, Austria), each well containing 200 µL of MH broth. Overnight cultures were adjusted to an OD_600_ of 0.6 (Amersham Biosciences, Little Chalfont, UK) and 200 µL of the bacterial suspension was added to each well, resulting in a starting antiseptic concentration of 64 µM in the first row. Plates were incubated overnight at 37 °C. MIC and corresponding sub-MIC values were determined in two independent experiments.

Based on these measurements, sub-MIC values of 2 µM CHX, 16 µM CPC, and 16 µM BAC were used for WT *E. coli*, whereas the adapted strains were exposed to their previously established sub-MICs of 3.9 µM CHX, 42.3 µM CPC, and 22.5 µM BAC, respectively.

### 2.3. Antiseptic Stress Exposure Experiments

To evaluate protein-level responses, WT and antiseptic-adapted *E. coli* strains were grown overnight in MH broth at 37 °C. WT bacteria were exposed to strain-specific sub-MIC concentrations of CHX, CPC, or BAC for 3 h at 37 °C. Adapted strains (CHX-P10, CPC-P10, BAC-P10) were incubated with their corresponding sub-MIC concentrations as determined above for 3 h at 37 °C. WT cultures incubated in fresh medium served as negative controls. Heat-shocked WT bacteria at 42 °C for 2 h served as positive controls.

### 2.4. Bacterial Lysis and Protein Quantification

For assessment of bacterial lysis and in order to determine conditions resulting in maximum protein yield, a single bacterial colony was inoculated in 5 mL of liquid medium, grown overnight at 37 °C and adjusted to an optical density OD_600_ of 1.0. The culture was centrifuged for 5 min at 15,000× *g* at 4 °C, the supernatant discarded and the pellet washed twice with Phosphate-Buffered Saline (PBS). After transfer to a 2 mL microcentrifuge tube and final centrifugation, the supernatant was removed. Next, 150 µL of CelLytic buffer (Merck KGaA, Darmstadt, Germany) and 150 mg beads were added and the suspension briefly mixed. Bacteria were then disrupted for 3, 5, or 10 min in pre-cooled adapters at 30 Hz in the bead mill. Subsequently, samples were centrifuged for 5 min at 15,000× *g* at 4 °C and the supernatants transferred to fresh tubes. To protect the proteins, 2 µL of protease inhibitor was added. Throughout all steps, lysates were kept on ice. Lysis assessment was performed in duplicate.

For protein quantification, a bovine serum albumin (BSA; Carl Roth GmbH und Co. KG, Karlsruhe, Germany) standard curve with increasing concentrations was prepared using a 96-well plate. Sample wells received a 1:5 dilution of the lysates containing 20 µL dH_2_O and 5 µL lysate. Both standards and samples were measured in duplicate. The assay reagents of the bicinchoninic acid (BCA) assay kit were mixed at a 1:50 ratio according to the manufacturer’s instructions, and 200 µL of solution was added to each well. The plate was briefly shaken and then incubated for 30 min at 37 °C under protection from light. Absorbance at 540 nm was measured using a spectrophotometer (Tecan Group Ltd., Männedorf, Switzerland), and protein concentrations were calculated from the standard curve. To enable reliable comparison of protein concentrations across conditions, all experiments were initiated with the same amount of bacterial biomass. For this purpose, 5 mL of bacterial culture adjusted to an OD_600_ of 1.0 was used as the standardized starting material for protein extraction.

### 2.5. SDS–PAGE and Coomassie Staining

A total of 10 µg protein per sample was mixed with Laemmli buffer, heated at 95 °C, and loaded onto 10% SDS–PAGE gels (Carl Roth GmbH & Co. KG, Karlsruhe, Germany). Electrophoresis was carried out at 70 V for 15 min followed by 110 V until the dye front reached the bottom. Gels were stained using 0.05% Coomassie solution (Thermo Fisher Scientific, Waltham, MA, USA) for 2 h and treated with destaining solution until protein bands were clearly visible.

### 2.6. Western Blot Analysis

First, 10 µg of protein was transferred to membranes for immunodetection. The stress-associated target proteins DnaK, GroEL, LexA, RecA, and OmpF were detected using specific primary antibodies (DnaK, GroEL, RecA: Enzo Life Sciences AG, Lausen, Switzerland; LexA: Bio-Techne, Minneapolis, MN, USA and OmpF: Biorbyt, Cambridge, UK) and secondary IgG antibodies (Enzo Life Sciences AG, Lausen, Switzerland). Chemiluminescent signal detection was performed using Clarity Western Enhanced Chemiluminescence (ECL)-Substrate or Clarity Max Western ECL-Substrate (Bio-Rad Laboratories GmbH, Feldkirchen, Germany). All Western blot experiments were performed in duplicate. Ponceau S staining served as a loading control. All findings reported in this study are based on relative protein abundance. Differences between experimental conditions therefore reflect proportional shifts in protein biomass within the cellular proteome, allowing comparison of up- or downregulated proteins.

### 2.7. Statistical Analyses

Median values and corresponding 25th and 75th percentiles of total protein concentration were calculated. Statistical analyses were performed using the Mann–Whitney U test (SPSS v29, IBM, Armonk, NY, USA). The significance level α was set to 0.05. Data visualization was performed using GraphPad Prism 10 (GraphPad Software, Boston, MA, USA).

## 3. Results

### 3.1. Bacterial Lysis and Protein Quantification

**Figure 1:**
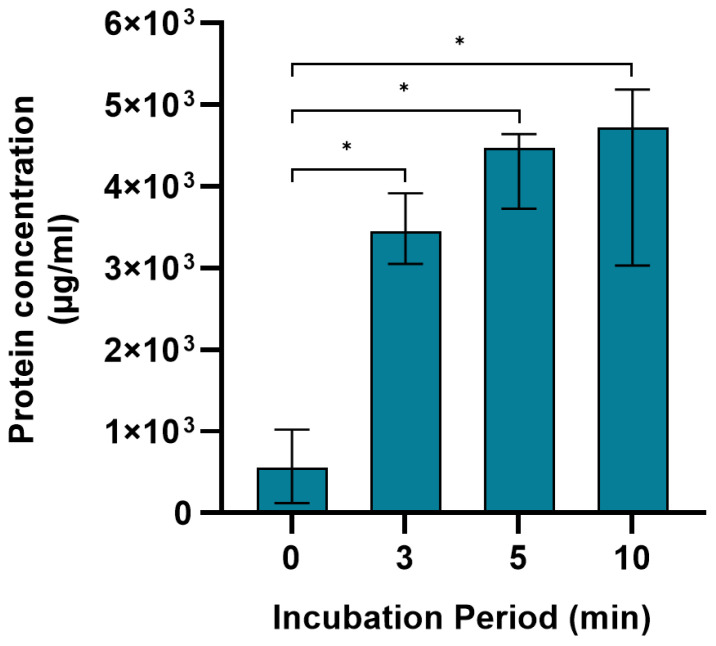
Results of *E. coli* lysis. Protein concentration (µg/mL) is plotted against incubation time (min). Samples were incubated in CelLytic buffer, with 0 min indicating conditions without mechanical disruption. Data are presented as median values and corresponding 25th and 75th percentiles. The number of independent samples per incubation period was *n* = 8. Asterisks indicate statistical significance (*p* ≤ 0.05).

Figure 1 shows the results of *E. coli* cell lysis in the bead mill. Comparisons between 0 min and all mechanically processed samples (3, 5 and 10 min) revealed highly significant differences (*p* < 0.001). Comparisons over time between 3 and 5 min, 3 and 10 min, 5 and 10 min were not significant. Consequently, CelLytic buffer and a bead mill shaking time of 5 min were employed for all further experiments including *E. coli* cell lysis.

### 3.2. MIC-Determination

Re-determination of MIC and sub-MIC values after thawing the archived *E. coli* P10 strains confirmed the values reported previously. For the wild-type strain, sub-MICs of 2 µM for CHX and 16 µM for both CPC and BAC were observed. Based on these findings, the following sub-MICs were used for subsequent stress exposure experiments: 2 µM CHX and 16 µM CPC and BAC for the wild-type strain, and 3.9 µM CHX, 42.3 µM CPC, and 22.5 µM BAC for the antiseptic-adapted strains.

### 3.3. Protein Expression Profiles

**Figure 2:**
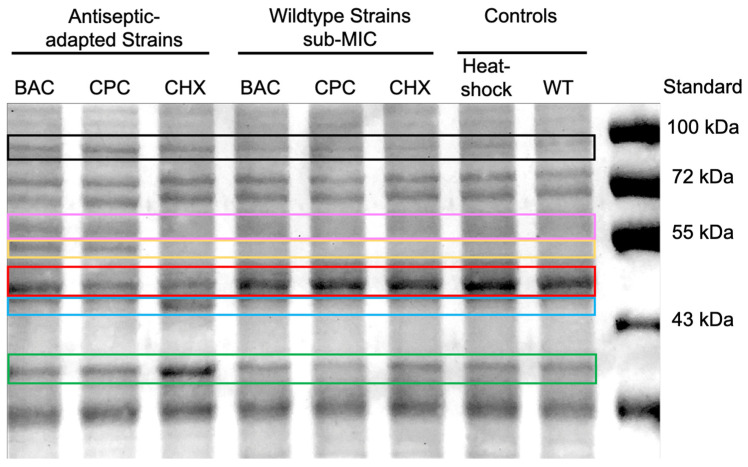
Protein expression profiles of antiseptic-adapted *E. coli* and wild-type *E. coli*. The rightmost lane shows the molecular weight standard in kilodaltons (kDa). WT = untreated wild type; heat shock = stress response induced at 42 °C for 2 h; wild-type strains sub-MIC = antiseptic-induced stress response for 3 h using the sub-MIC values determined for wild-type *E. coli*; antiseptic-adapted strains = antiseptic-induced stress response for 3 h using sub-MIC values reported by Schwarz et al. [20]; BAC = benzalkonium chloride; CPC = cetylpyridinium chloride; CHX = chlorhexidine digluconate; black box = increased protein expression at approximately 100 kDa in antiseptic-adapted strains; purple and yellow boxes = changes around 55 kDa in BAC- and CPC-adapted strains; green box = increased expression below 43 kDa in CHX-adapted strains; blue box = additional bands between 43 and 55 kDa in CHX-adapted strains; red box = reduced protein expression between 43 and 55 kDa in antiseptic-adapted strains.

Figure 2 presents the results of the Coomassie staining of *E. coli* proteins. The SDS gel reveals an upregulation of protein expression at approximately 100 kDa in the antiseptic-adapted strains compared with the untreated wild type and the sub-MIC-treated wild-type *E. coli* (black box). Additional changes were detected at 55 kDa in the BAC- and CPC-adapted strains compared to the untreated wild type and the sub-MIC–treated wild-type *E. coli* (purple and yellow boxes). A further upregulation was observed in the CHX-adapted *E. coli* strain below 43 kDa (green box). An additional band between 55 and 43 kDa was observed in the CHX-adapted strain compared to the sub-MIC-treated wild type and the untreated wild type (blue box). In the same region, a downregulation of protein expression was evident in the antiseptic-adapted strains (red box).

### 3.4. Western Blot

**Figure 3:**
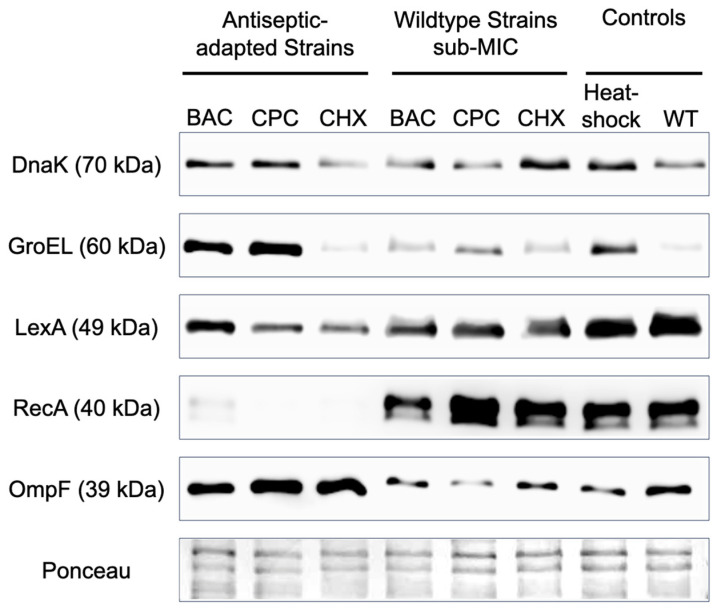
Protein expression profiles in antiseptic-adapted *E. coli*, wild-type *E. coli* exposed to sub-MIC levels, and control groups including heat-shocked and untreated wild-type *E. coli*. Samples were run on different gels. WT = untreated wild type; heat shock = stress response induced at 42 °C for 2 h; wild-type strains sub-MIC = antiseptic-induced stress response for 3 h using sub-MIC values determined for wild-type *E. coli*; antiseptic-adapted strains = antiseptic-induced stress response for 3 h using sub-MIC values reported by Schwarz et al. [15]. Increased band intensity of DnaK and GroEL is observed in heat-shocked and antiseptic-adapted strains, whereas LexA and RecA show reduced expression in antiseptic-adapted strains. OmpF expression is decreased under acute stress conditions but increased in adapted strains. Ponceau S staining served as a loading control to confirm equal protein loading across lanes.

Figure 3 shows the Western blots with the protein expression profiles of antiseptic-adapted *E. coli* vs. wild-type *E. coli*. Uncropped full original chemiluminescence scans are provided in Appendix A. For DnaK, wild-type *E. coli* exposed to heat shock (positive control) exhibited upregulation compared to the untreated wild type. Upregulation was further observed in the wild type treated with the CHX sub-MIC, as well as in the BAC- and CPC-adapted strains, compared to the untreated wild type. For GroEL, the heat shock control exhibited upregulation compared to the untreated wild type. The BAC- and CPC-adapted strains exhibited GroEL upregulation compared to the untreated wild type. For the remaining bands, a slight upregulation was observed. In the BAC- and CPC-adapted strains, the increase in GroEL expression correlated with the upregulation of DnaK. LexA expression was highest in the untreated wild type. A slight decrease was observed in the heat shock control, whereas the remaining bands showed a stronger reduction compared to the wild type. CHX- and CPC-adapted strains exhibited reduced LexA expression relative to the untreated wild type. RecA expression was highest in the untreated wild type, with similar expression levels in the heat shock control and in the sub-MIC–treated wild type. In contrast, RecA expression in the antiseptic-adapted strains was lower with barely detectable bands. OmpF expression in the heat shock control and in the sub-MIC-treated wild-type strains was reduced compared to the untreated wild type. However, in the antiseptic-adapted strains, OmpF expression was elevated compared to the wild type.

## 4. Discussion

The aim of this study was to investigate protein-level changes in *E. coli* wild-type and antiseptic-adapted strains following exposure to subinhibitory concentrations of CHX, CPC and BAC. Treatment with antiseptics at subinhibitory concentrations induces metabolic changes since external stress factors may trigger the up- or downregulation of proteins in order to increase cellular survival [11,13,15,20]. For the antiseptic-adapted strain *E. coli* ATCC 25922, a preceding study by Schwarz et al. described phenotypic adaptation as shown by an increase in MIC values compared to *E. coli* wild type strain after ten passages (P10) in subinhibitory concentrations of antiseptics, resulting in CHX-P10, CPC-P10 and BAC-P10 strains with sub-MIC values of 3.9 µM for CHX, 42.3 µM for CPC, and 22.5 µM for BAC [20]. In the current publication, heat shock was included as a methodological control to benchmark the capacity of bacteria to react to stress responses. While both subinhibitory antiseptic exposure and heat shock act as stress conditions that trigger adaptive changes in protein expression, they are perceived differently by bacterial cells and therefore elicit distinct cellular responses. Accordingly, heat shock was used as a reference condition to confirm the induction of stress-associated protein expression rather than as a directly comparable model of antiseptic-induced stress. Heat-shocked wild-type *E. coli* showed higher DnaK expression compared with the untreated wild type, as DnaK acts as a heat shock protein, protecting cellular proteins from thermal denaturation [26,28]. Exposure of wild-type bacteria to CHX at sub-MIC levels resulted in similarly increased DnaK expression, indicating that antiseptic stress can induce similar protective mechanisms. However, heat and antiseptic stressors induce distinct, partially overlapping cellular responses but cannot be regarded as biologically equivalent [26,28].

Antiseptic-adapted *E. coli* strains demonstrated distinct protein expression profiles consistent with long-term adaptation rather than acute stress perception. Specifically, CPC- and BAC-adapted strains showed clear upregulation of DnaK and GroEL. Because DnaK supports GroEL activation and both act together to stabilize and refold proteins under stress [29,30,31], their concurrent upregulation suggests an enhanced capacity for protein protection in adapted strains. Repeated sub-MIC exposure during the passaging process [20] may prime these strains to respond more efficiently to renewed antiseptic stress. Similar observations have been made in CHX-adapted Streptococcus spp. on the gene expression level, where groEL was consistently upregulated [15], supporting the idea that protein protection pathways represent a common mechanism during antiseptic adaptation.

In contrast, DnaK levels in CPC- and BAC-treated wild-type bacteria and in CHX-treated adapted strains were similar to those of untreated wild-type *E. coli*. This suggests that the stress response may be transient under these conditions, that DnaK expression had already declined after 3 h of incubation or that other proteins mediate adaptation in these strains.

Interestingly, the expression patterns differed between the antiseptics. While CPC- and BAC-adapted strains exhibited a pronounced upregulation of both DnaK and GroEL, this response was less evident in CHX-adapted *E. coli*. One possible explanation is the different mechanisms of action of the investigated antiseptics. CPC and BAC belong to the quaternary ammonium compounds and primarily disrupt bacterial membranes, resulting in an increased demand for chaperone-mediated protein folding and stabilization induced by DnaK and GroEL. In contrast, CHX may additionally induce cytoplasmic coagulation and protein precipitation, suggesting a broader impact on intracellular protein homeostasis, which may promote alternative adaptive responses [32]. Although these hypothesized mechanisms remain to be confirmed experimentally, they may contribute to the distinct protein expression profiles observed in the present study.

Antiseptic adaptation also involved pronounced alterations in DNA damage response pathways. Antiseptic-adapted *E. coli* strains exhibited reduced RecA expression accompanied by decreased LexA levels. Since RecA promotes LexA autoproteolysis and thereby activates the SOS response [25,33,34], the concurrent reduction of both proteins suggests a fundamentally altered regulation of SOS signalling in adapted strains. One possible explanation is that long-term antiseptic adaptation reduces intracellular damage upon renewed exposure, thereby diminishing the requirement for RecA-mediated DNA repair. Alternatively, repeated subinhibitory stress may redirect SOS regulation toward constitutive protective mechanisms rather than acute induction [3,35].

Modulation of DNA repair–associated pathways has been described in CHX-adapted *Streptococcus* spp. on the gene expression level, where components of the RecBCD system, including recD, were differentially regulated following adaptation [15]. Together, these observations indicate that downmodulation of the SOS response may represent a common adaptive strategy during prolonged exposure to antiseptics, contributing to increased tolerance by limiting high-effort stress-induced repair processes.

Changes in OmpF expression further point to the involvement of membrane-associated mechanisms. Sub-MIC treatment and heat shock reduced OmpF levels in wild-type bacteria, consistent with prior studies showing that porin downregulation enhances membrane stability under stress [36]. Conversely, antiseptic-adapted strains showed elevated OmpF levels, which may reflect altered membrane permeability or modified interactions with cationic antiseptics like CHX and CPC. Increased or structurally altered porins can influence efflux and influx dynamics, shaping susceptibility to various antimicrobials [4,27,37].

The significant increase in protein concentration between non-disrupted samples and mechanically processed samples confirms the substantial contribution of mechanical lysis to protein extraction. However, the absence of significant differences between shaking durations beyond 3 min, particularly between 5 and 10 min, indicates that protein recovery rapidly reaches a plateau. This supports the selection of a 5-min lysis time as a suitable compromise between efficient protein extraction and preservation of protein integrity, minimizing potential heat-induced denaturation without compromising yield.

Overall, the data indicate that repeated exposure to subinhibitory concentrations of cationic antiseptics leads to multifactorial protein-level adaptations of *E. coli*. These involve changes in chaperone systems, DNA-damage regulators, and membrane-associated factors, supporting that reduced susceptibility to antiseptics arises from several protective mechanisms rather than from a single pathway. This interpretation is consistent with the findings of Muehler et al., who showed on the transcriptome level that sublethal CHX exposure in S. mutans activated a broad stress response affecting transport processes, oxidative protection pathways and DNA-repair functions [13]. Their observation of differentially regulated DNA-repair genes corresponds with the altered RecA to LexA balance observed in our study, suggesting that modulation of SOS signalling may represent a common adaptive pattern during repeated exposure to cationic antiseptics. Likewise, the strong induction of chaperone networks reported in S. mutans is consistent with the upregulation of DnaK and GroEL in the adapted *E. coli* strains used in this study, indicating that increased proteostasis capacity may be a shared strategy across different bacterial species. The phenotype-level observations of Schwarz et al. [20], including reduced antiseptic susceptibility and altered protein patterns after repeated sub-MIC exposure, also become more comprehensible when considered alongside our findings, as we identified specific stress-associated proteins that consistently differ between wild-type and adapted strains. The reduced RecA and LexA levels detected here further suggest that reduced antiseptic susceptibility is not only linked to changes in membrane properties and overall protein expression but also involves adjustments in DNA-damage regulation. The present findings gain particular clinical relevance when considering the conditions under which antiseptics are used in routine practice. Although CHX, CPC and BAC are generally applied at concentrations substantially exceeding the MIC, residual antiseptic concentrations may occur following dilution by biological fluids, adsorption to organic material, or persistence on treated surfaces, thereby exposing bacteria to sub-inhibitory concentrations [16,17]. Such sub-MIC exposure has been shown to permit bacterial survival and to induce metabolic adaptations, transient reductions in antiseptic susceptibility, and stable cross-resistance to clinically relevant antibiotics [16]. Moreover, repeated exposure to sub-MIC CHX concentrations has been demonstrated to select stable resistant mutants characterized by altered outer membrane permeability, increased efflux pump activity and reduced susceptibility to multiple antibiotic classes, providing further evidence that prolonged sub-inhibitory antiseptic exposure represents a relevant selective pressure [38]. The protein-level alterations observed in the present study, including changes in stress response proteins, DNA damage regulators, and membrane-associated proteins, may therefore represent molecular mechanisms contributing to these adaptive phenotypes. However, unlike for antibiotics, it remains unknown whether maintaining antiseptic concentrations above the MIC is sufficient to prevent the development of adaptive responses, as validated mutant prevention concentration (MPC) and mutant selection window (MSW) thresholds have not yet been established for antiseptics [18]. Consequently, further studies are required to define clinically relevant concentration thresholds that reliably prevent the selection of antiseptic-adapted bacterial populations [18].

The antiseptic-adapted *E. coli* strains analysed in the present study were generated and phenotypically characterized in our previous study by Schwarz et al., which demonstrated stable adaptation following repeated exposure to sub-MIC concentrations of CHX, CPC and BAC [20]. Whereas that study primarily focused on phenotypic adaptation, including changes in MICs and alterations in overall protein banding patterns, the present study extends these findings by identifying specific stress-associated proteins that are differentially regulated in the adapted strains.

The present study extends our previous work by providing the first targeted analysis of stress-associated protein expression in antiseptic-adapted *E. coli*. Whereas earlier studies demonstrated stable adaptation following repeated exposure to sub-MICs and described changes in antimicrobial susceptibility as well as transcriptomic alterations associated with long-term adaptation, including differential regulation of stress response pathways, transport systems and metabolic processes, the present investigation identifies specific stress-associated proteins that are differentially regulated in adapted strains [8,13,15,20]. By linking the previously observed phenotypic adaptation to defined molecular alterations, this study provides novel insight into the biological processes underlying antiseptic adaptation. These findings establish a foundation for future investigations exploring the relationship between proteomic alterations, reduced antiseptic susceptibility and the potential development of cross-resistance.

Taken together, these findings emphasize that antiseptic adaptation is a complex and multilayered process and underline the need for more standardized methods to allow better comparison across transcriptomic, proteomic, and phenotypic studies and to clarify its implications for antimicrobial resistance development.

## 5. Conclusions

This study demonstrates that repeated exposure of *E. coli* to subinhibitory concentrations of commonly used antiseptics results in alterations in protein expression profiles. The differential regulation of key stress-associated proteins, including DnaK, GroEL, LexA, RecA and OmpF, provides strong evidence for protein-level changes contributing to phenotypic adaptation. Further investigations at both the protein and gene expression levels are necessary to fully elucidate the mechanisms involved and to assess the potential implications for resistance and cross-resistance development.

## Figures and Tables

**Figure A1:**
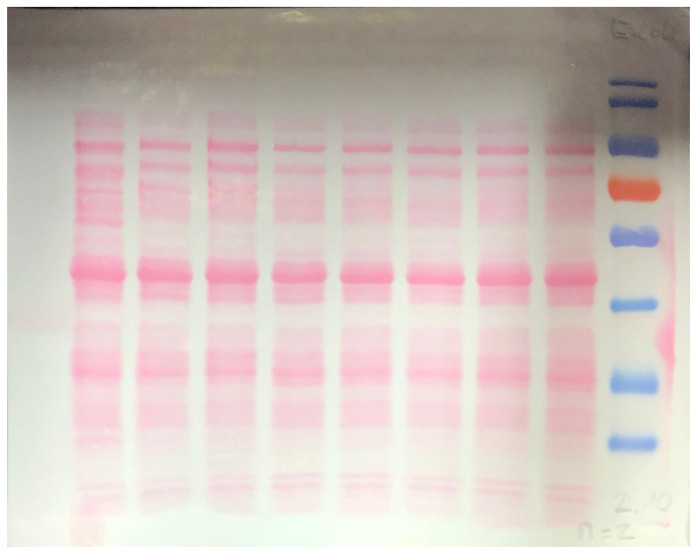
Unprocessed Coomassie staining of *E. coli* proteins.

**Figure A2:**
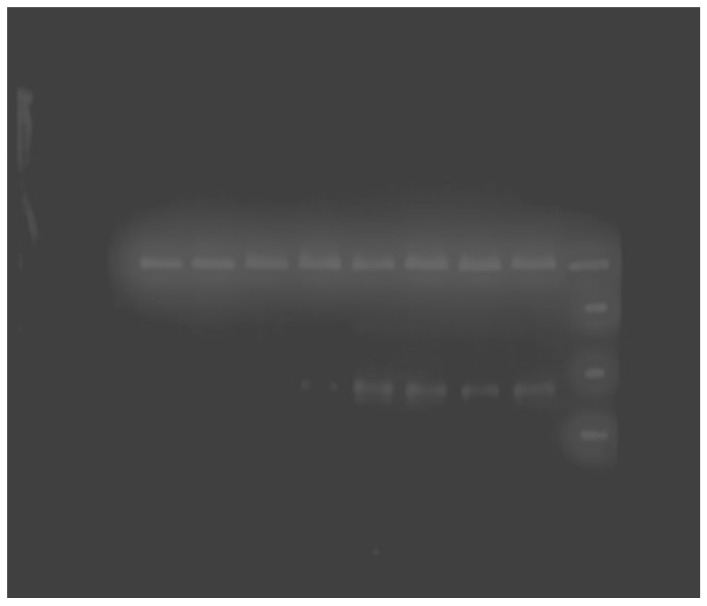
Representative unprocessed chemiluminescence Western blot scan of DnaK.

**Figure A3:**
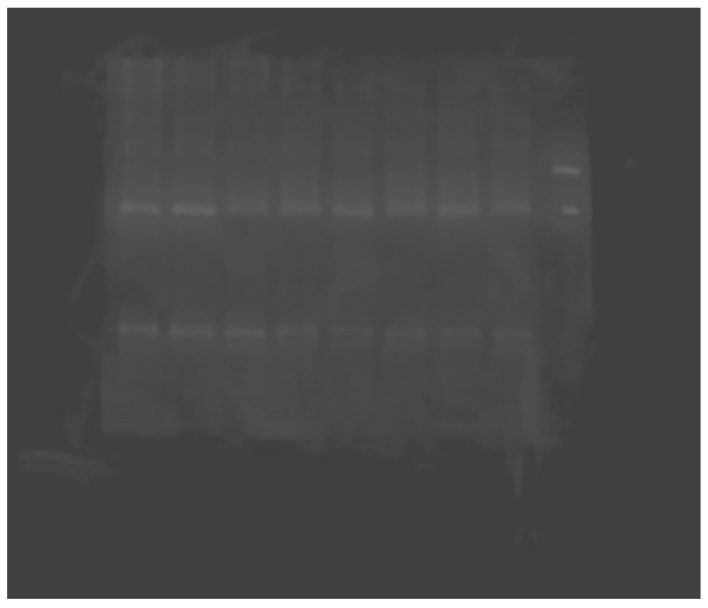
Representative unprocessed chemiluminescence Western blot scan of GoEL.

**Figure A4:**
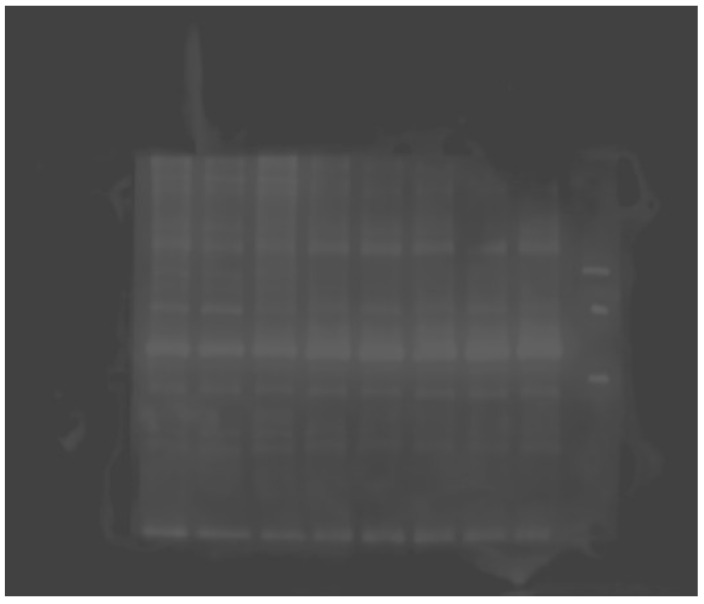
Representative unprocessed chemiluminescence Western blot scan of LexA.

**Figure A5:**
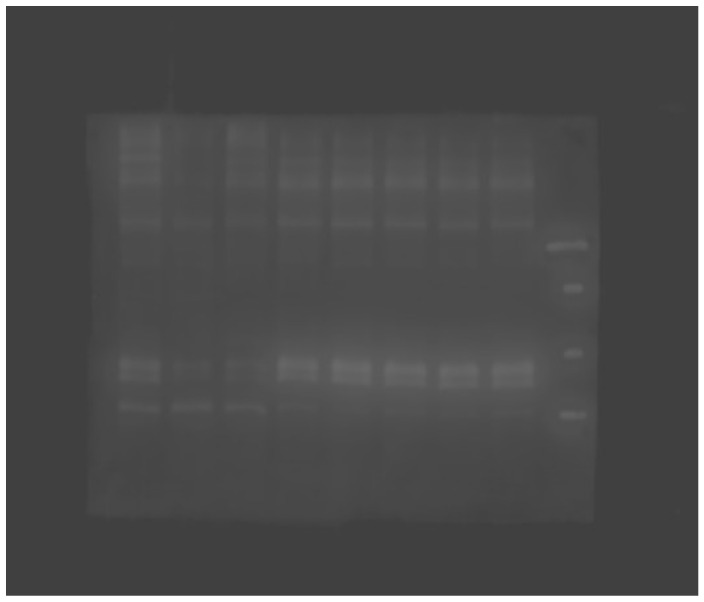
Representative unprocessed chemiluminescence Western blot scan of RecA.

**Figure A6:**
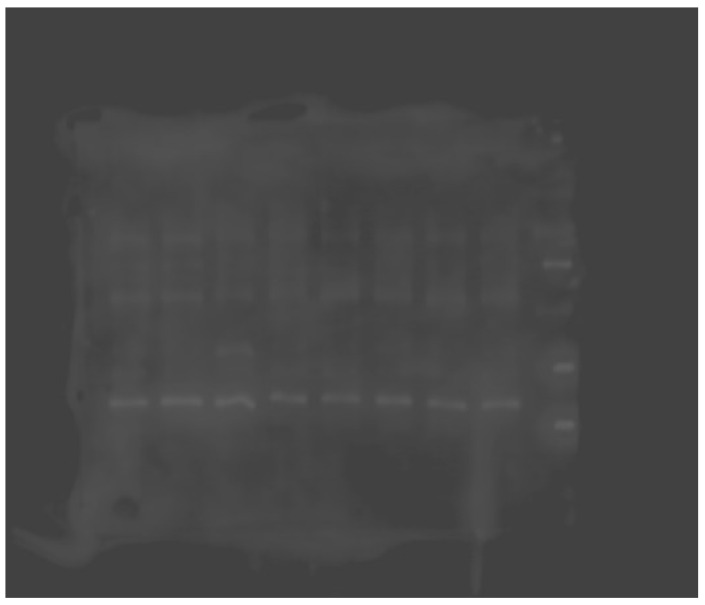
Representative unprocessed chemiluminescence Western blot scan of OmpF.

## Data Availability

The datasets used or analyzed during this study are available from the corresponding author upon reasonable request.

## Abbreviations

The following abbreviations are used in this manuscript:

AMR	Antimicrobial Resistance
ATCC	American Type Culture Collection
BAC	Benzalkonium Chloride
BCA	Bicinchoninic Acid
BSA	Bovine Serum Albumin
CHX	Chlorhexidine
CPC	Cetylpyridinium Chloride
DFG	German Research Foundation (Deutsche Forschungsgemeinschaft)
DNA	Deoxyribonucleic Acid
DnaK	Dna K chaperone protein
ECL	Enhanced Chemiluminescence
*E. coli*	*Escherichia coli*
GBD	Global Burden of Disease
GroEL	Growth-related protein E large subunit
h	Hours
IADR	International Association for Dental, Oral and Craniofacial Research
IgG	Immunoglobulin G
kDa	Kilodalton
LexA	LexA repressor protein
MH	Müller–Hinton Broth
MIC	Minimum Inhibitory Concentration
OD600	Optical Density at 600 nm
OmpF	Outer Membrane Protein F
PBS	Phosphate-Buffered Saline
RecA	Recombinase A
SDS-PAGE	Sodium Dodecyl Sulfate Polyacrylamide Gel Electrophoresis
SOS	SOS Response
sub-MIC	Sub-Minimum Inhibitory Concentration
WT	Wild Type
